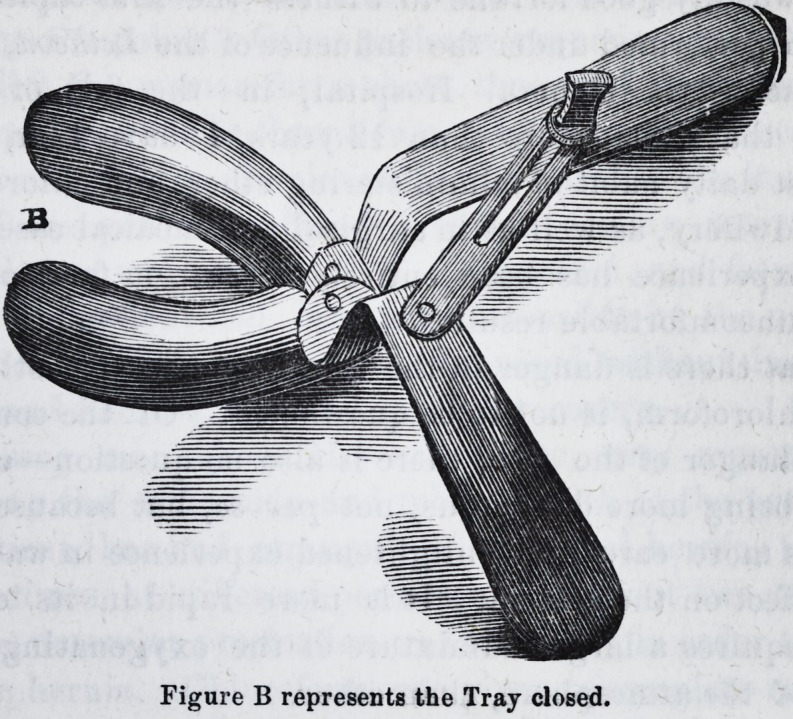# An Improved Model Tray

**Published:** 1859-07

**Authors:** J. Humby


### ARTICLE XI.

*An Improved Model Tray.* By MR. J. HUMBY.

HAVING frequently experienced considerable trouble in procuring a correct impression of the alveolar ridge of the lower jaw, especially in cases where the width at the coronoid processes is very great and the mouth unusually small, I have perfectly succeeded in overcoming these difficulties by an improved model tray, a description of which, with drawings, I beg to forward for publication in the "Dental Review," believing that upon trial it will be found as useful in the practice of every dentist as it has been in mine.

Figure A represents the Tray opened.

1, 2. A plate struck up to a lower (plain) model: then sawn in two with a V piece cut from the centre of the inside, to allow the Tray to nearly close.

3. The handles bent so that the part near the mouth may clear the lips in taking the impression.

4. The slide, with a thumb-screw, to fix the handles when the Tray is adjusted to the required size.

5. The thumb-screw.

The material best adapted for taking impressions in this tray is that prepared by Mr. Stent, of Coventry street, and called by him his improved plastic ivory. I prefer this composition on account of its great tenacity, while its contractibility being much less than that of wax, the tray can be opened or closed when in the mouth without injury to

B

Figure B represents the Tray closed.

the impression. The composition should be turned over the edge of the Tray which holds it thereon, by which means a correct impression is obtained. I have tried several contrivances, and am convinced that after a dentist has used this new and, I may say, valuable tray, it will be evident to him that difficult impressions may be taken with greater accuracy than can be obtained by any other means.

Mr. Jack, jun., surgical instrument maker, has made these Trays according to instructions, and to my entire satisfaction.—*London Dent. Review.*

East-road, City-road, Dec. 1858.

## Figures and Tables

**Figure A f1:**
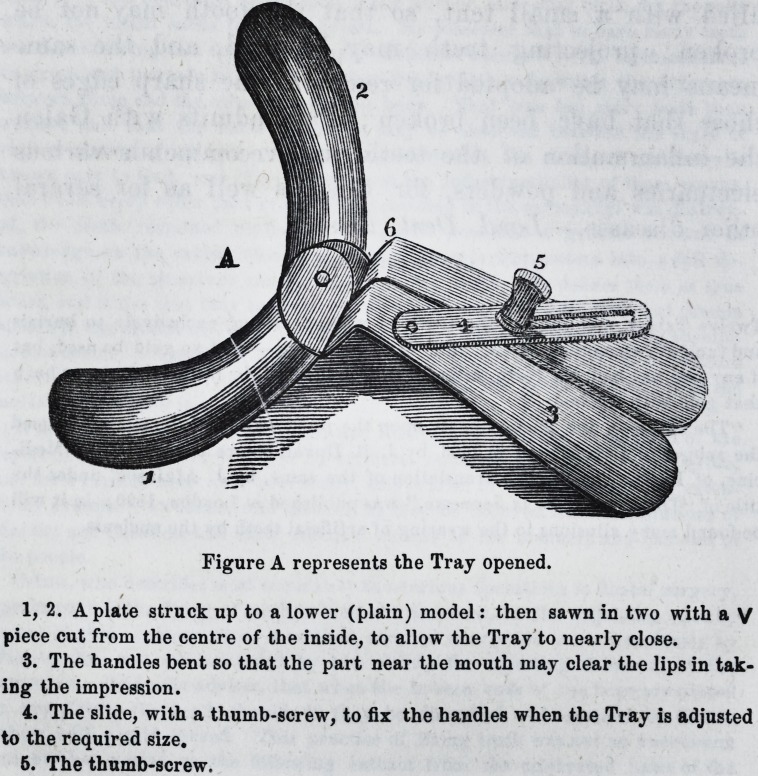


**Figure B f2:**